# In vitro fertilization–embryo transfer resulting in a live birth in a woman with benign multicystic peritoneal mesothelioma: A case report

**DOI:** 10.20407/fmj.2024-036

**Published:** 2025-08-06

**Authors:** Kiriko Kotani, Eiji Nishio, Shota Oikawa, Miho Ishikawa, Eriko Sakakibara, Arata Kobayashi, Hironori Miyamura, Haruki Nishizawa

**Affiliations:** 1 Department of Obstetrics and Gynecology, Fujita Health University, School of Medicine, Toyoake, Aichi, Japan; 2 Department of Clinical Laboratory, Fujita Health University Hospital, Toyoake, Aichi, Japan

**Keywords:** Benign multicystic peritoneal mesothelioma, Delivery, Immunohistochemical analysis, In vitro fertilization

## Abstract

We here present what we believe to be the first documented successful pregnancy and live birth following in vitro fertilization (IVF) and embryo transfer in a patient with benign multicystic peritoneal mesothelioma (BMPM). BMPM, a rare peritoneal disease, predominantly affects women of reproductive age. This condition is thought to result from peritoneal irritation, which may be exacerbated by invasive procedures such as oocyte retrieval during IVF. In this report, we present data on a 28-year-old woman who was diagnosed with BMPM by laparoscopic biopsy performed to investigate abdominal pain. Follow-up imaging during and after the pregnancy showed no progression of the cysts. The patient gave birth to a 3130-g baby by vaginal delivery at 40 weeks gestation. An MRI was performed one month after delivery; the findings resembled those of previous MRIs, except for the presence of multiple cysts in the omentum. To the best of our knowledge, there have been no published reports of infertility treatments, pregnancy, or childbirth in women with BMPM. Thus, careful follow-up, including regular MRI scans, is required.

## Introduction

Malignant tumor dissemination and pseudomyxoma peritonei (i.e., cysts in multiple locations in the abdominal cavity) are rare. Causes include intra-abdominal miliary tuberculosis, lymphangioma, and benign multicystic peritoneal mesothelioma (BMPM).^1^ BMPM, a rare peritoneal condition, occurs during a woman’s reproductive years and results from changes in mesothelial cells in response to peritoneal irritation. To date, approximately 200 cases have been reported.^2^ Although this condition is benign, malignant transformation has been reported in some cases; therefore, careful follow-up is required.^3^ Oocyte retrieval, an invasive procedure, involves puncturing the ovary with a needle through the vagina. Given that BMPM is believed to occur in response to peritoneal irritation, caution should be exercised when performing oocyte retrieval as this procedure may aggravate the condition. To the best of our knowledge, no reports of in vitro fertilization (IVF) or successful pregnancies in patients with BMPM have been published so far. We here report a patient with BMPM who underwent IVF and embryo transfer, became pregnant, and achieved a live birth.

## Case Presentation

**Figure 1:**
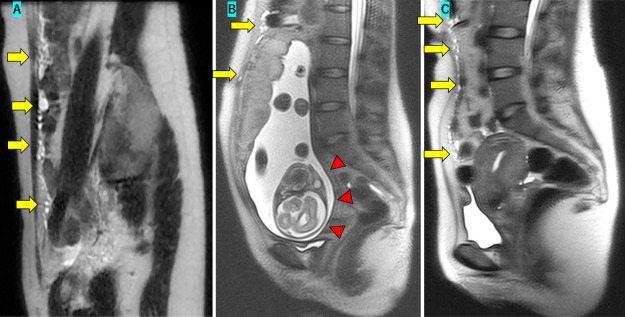
A MRI image on presentation showing multiple cysts (arrows) in the omentum, B MRI image obtained at 28 weeks’ gestation showing multiple cysts (arrows) and a fetus (triangle), C MRI image obtained one month after delivery showing multiple cysts (arrow), as in previous MRIs

**Figure 2:**
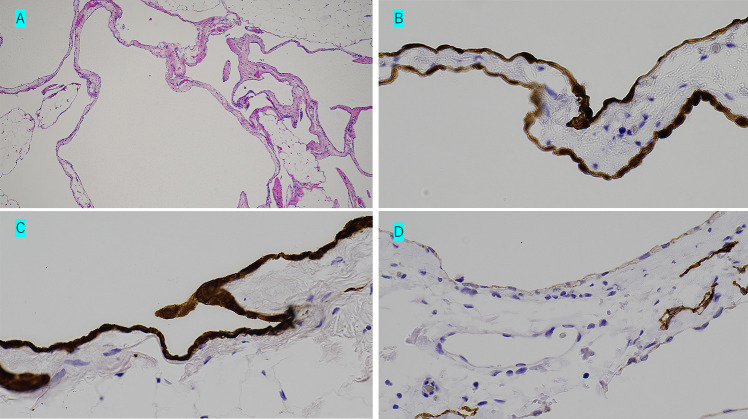
A Photomicrograph of segment of omentum showing multiple benign mesothelial cysts (hematoxylin and eosin stain, original magnification ×100), B Photomicrograph of segment of omentum (immunohistochemical stain, positive for calretinin, original magnification ×400), C Photomicrograph of segment of omentum (immunohistochemical stain, positive for cytokeratin, original magnification ×400), D Photomicrograph of segment of omentum (immunohistochemical stain, negative for D2-40, original magnification ×400)

Our patient was a 28-year-old nulliparous woman. She had consulted her family doctor regarding help with conceiving and had failed to achieve conception during four rounds of artificial insemination. She then presented to an emergency department with the chief complaint of abdominal pain. Magnetic resonance imaging (MRI) (Figure 1A) revealed multiple nodules in the omentum. Subsequent upper and lower endoscopy and small bowel capsule endoscopy failed to reveal a cause for the nodules. A diagnostic laparoscopy revealed multiple nodules in the omentum and pelvic floor. Part of the omentum was removed, histological examination of which resulted in identification of multiple benign mesothelial cysts (Figure 2A).

On immunohistochemical analysis, the cells were diffusely positive for calretinin (Figure 2B) and cytokeratin (Figure 2C) and negative for D2-40 (Figure 2D). A T-SPOT test was negative, ruling out tuberculosis. The locations of the lesions precluded complete resection, and the patient was followed up with MRI imaging. After informing the patient that there were no reports of pregnancy in women with her condition and that the effects of infertility treatments were unknown, she insisted nonetheless that we perform IVF in a further attempt to achieve a pregnancy. IVF and embryo transfer at our hospital were successful, the pregnancy progressed smoothly, and an MRI performed at 28 weeks’ gestation (Figure 1B) showed no increase in the cyst sizes. The patient gave birth to a 3130-g baby by vaginal delivery at 40 weeks gestation. An MRI performed one month after delivery showed similar findings as previous examinations, except for the presence of multiple cysts in the omentum (Figure 1C).

## Discussion

Multiple cysts in the abdominal cavity can be attributable to malignant tumor dissemination, pseudomyxoma peritonei, lymphangioma, peritoneal tuberculosis, and many other conditions. Chronic peritonitis is reportedly associated with proliferation and migration of peritoneal cells in the surrounding areas and is often associated with degeneration of the underlying connective tissue.^4,5^ It occurs more commonly in relatively young women (mean age 37 years). Proposed risk factors include previous surgery, endometriosis, and pelvic peritonitis.^6^ In the present case, a left chocolate cyst was identified on an MRI. Most patients with BMPM are reportedly asymptomatic until the lesions spread to other organs; however, our patient presented with abdominal pain. BMPM is difficult to diagnose by imaging, immunohistochemistry usually being required. In our case, T-SPOT was negative, which excluded peritoneal tuberculosis, and immunohistochemistry was negative for D2-40, which excluded lymphangioma. Oocytes are retrieved by puncturing the ovaries through the vagina. To date, there is no evidence that IVF, pregnancy, or childbirth promote progression of BMPM lesions; however, caution is warranted.

Three hypotheses regarding development of BMPM have been proposed.^7^ The first is that it results from chronic inflammation caused by previous surgery, endometriosis, pelvic inflammatory disease, or peritoneal dialysis.^6^ Our patient had a chocolate cyst, which is consistent with this hypothesis. The second hypothesis is that BMPM results from noninflammatory, purely neoplastic, transformation of the peritoneal mesothelium.^8^ A neoplastic origin has also been proposed on the basis of the characteristic slow, progressive growth of untreated lesions, marked tendency for recurrence after surgical resection, low incidence of previous abdominal infections, and high disease-related mortality.^9^ The third is the hormonal hypothesis, which asserts that the development and progression of BMPM is closely related to sensitivity to sex hormones.^10^ Given that our patient was in her 20s and of reproductive age, estrogen may have been involved in the pathogenesis of her condition. Thus, there is a theoretical concern that the continued high concentrations of estrogen associated with infertility treatment and pregnancy may exacerbate BMPM. Given that there have been no published reports of infertility treatments, pregnancy, or childbirth in women with BMPM, careful follow-up, including regular MRI scans, of such patients is required.

## Data Availability

The authors confirm that the data supporting the findings of this study are available within the article [and/or] its supplementary materials.
